# Identification of high-risk habitats of *Oncomelania hupensis*, the intermediate host of *schistosoma japonium* in the Poyang Lake region, China: A spatial and ecological analysis

**DOI:** 10.1371/journal.pntd.0007386

**Published:** 2019-06-17

**Authors:** Congcong Xia, Yi Hu, Michael P. Ward, Henry Lynn, Si Li, Jun Zhang, Jian Hu, Shuang Xiao, Chengfang Lu, Shizhu Li, Ying Liu, Zhijie Zhang

**Affiliations:** 1 Department of Epidemiology and Biostatistics, School of Public Health, Fudan University, Shanghai, P. R. China; 2 Key Laboratory of Public Health Safety, Ministry of Education, Shanghai, P. R. China; 3 Collaborative Innovation Center of Social Risks Governance in Health, School of Public Health, Fudan University, Shanghai, P. R. China; 4 Department of Infection Control Administration, Fudan University Shanghai Cancer Center, Department of Oncology, Shanghai Medical College, Fudan University, Shanghai, P. R. China; 5 Sydney School of Veterinary Science, The University of Sydney, Sydney, Australia; 6 Key Laboratory of Poyang Lake Wetland and Watershed Research, Ministry of Education, Jiangxi Normal University, Nanchang, P. R. China; 7 National Institute of Parasitic Diseases, Chinese Center for Disease Control and Prevention, Key Laboratory of Parasite and Vector Biology, Ministry of Health, Shanghai, P. R. China; University of Florida, UNITED STATES

## Abstract

**Background:**

Identifying and eliminating snail habitats is the key measure for schistosomiasis control, critical for the nationwide strategy of eliminating schistosomiasis in China. Here, our aim was to construct a new analytical framework to predict high-risk snail habitats based on a large sample field survey for *Oncomelania hupensis*, providing guidance for schistosomiasis control and prevention.

**Methodology/Principal findings:**

Ten ecological models were constructed based on the occurrence data of *Oncomelania hupensis* and a range of variables in the Poyang Lake region of China, including four presence-only models (Maximum Entropy Models, Genetic Algorithm for rule-set Production, Bioclim and Domain) and six presence-absence models (Generalized Linear Models, Multivariate Adaptive Regression Splines, Flexible Discriminant Analysis, as well as machine algorithmic models–Random Forest, Classification Tree Analysis, Generalized Boosted Model), to predict high-risk snail habitats. Based on overall predictive performance, we found Presence-absence models outperformed the presence-only models and the models based on machine learning algorithms of classification trees showed the highest accuracy. The highest risk was located in the watershed of the River Fu in Yugan County, as well as the watershed of the River Gan and the River Xiu in Xingzi County, covering an area of 52.3 km^2^. The other high-risk areas for both snail habitats and schistosomiasis were mainly concentrated at the confluence of Poyang Lake and its five main tributaries.

**Conclusions/Significance:**

This study developed a new distribution map of snail habitats in the Poyang Lake region, and demonstrated the critical role of ecological models in risk assessment to directing local field investigation of *Oncomelania hupensis*. Moreover, this study could also contribute to the development of effective strategies to prevent further spread of schistosomiasis from endemic areas to non-endemic areas.

## Author summary

*Oncomelania hupensis* is the sole intermediate host of *Schistosoma japonicum* and it is critical for the long-term sustainable control and elimination of schistosomiasis. We suggest a new analytical framework to predict high-risk snail habitats based on ecological models. Their predictive performance were compared via three indices (receiver-operating characteristic [ROC] curve, Kappa value and calibrating plot) and further they were classified into three grades with different discrimination ability. The four best models were chosen and combined to identify snail habitats, facilitating classification of the snail habitats in the Poyang Lake region into four risk grades, which should be prioritized for interruption of the snail life cycle, with control intensity and scope dependent on the available health resources.

## Introduction

Schistosomiasis japonica, a water-borne parasitic disease, has long been prevalent in 12 provinces located along the Yangtze River in China. It is caused by infection with the parasite *Schistosoma japonicum*, which has a unique intermediate host, *Oncomelania hupensis*. During the past 60 years, China has made remarkable progress towards eliminating schistosomiasis japonica. Much of this progress was made during the World Bank Loan Project (1992–2001), e.g., prevalence was reduced by more than 50%[1]. Additional control activities were initiated in 2005 based on replacement of draft cattle with mechanisation and increased health education, which resulted in further gains in controlling this disease. Up to 2015, 12 endemic provinces located along the Yangtze River had reached the stage of transmission being interrupted or controlled[2]. Despite declining schistosomiasis prevalence year-by-year and an overall low prevalence, this disease remains a serious problem in China. Transmission risk remains high in some regional areas, especially the lake and marshland regions. It is alarming that the area of snail habitat in some regions (e.g. Hubei, Hunan, Jiangxi and Anhui) are still extensive and new or infective snail habitats are still being discovered, especially in these lake and marshland regions (e.g. Dongting Lake and Poyang Lake regions)[3]. This represents a serious challenge for preventing a rebound in prevalence and spread of schistosomiasis in China. Therefore, how to predict high-risk areas accurately and allocate the limited health resources effectively has become the key and future focus of schistosomiasis prevention and control activities.

It is widely acknowledged that the epidemiology of *S*. *japonicum* infections has a particular spatial characteristic because it depends on the presence of its sole intermediate host snail, *O*.*hupensis*, whose reproduction is governed by specific climatic and environmental condition[4]. It is certain that where schistosomiasis occurs (excluding imported cases), *O*.*hupensis* exist. Therefore, controlling the spread of *O*.*hupensis*, especially infected hosts, will lead to long-term effective schistosomiasis control since killing and eliminating snail habitats is the most sustainable measure for controlling this disease. However, this is challenging from the perspective of a national control strategy because of the massive investment of manpower, physical and financial resources, and potential environmental pollution.

Knowledge about a species’ ecological and geographical distribution is of great concern in fields such as ecological conservation and species biodiversity. To assess the spatial distribution of *O*.*hupensis*, field investigations have been widely applied. However, for many regions (including mountains and swamp), detailed and accurate data on spatial distribution of snail habitats are not available because collecting such data via snail surveys is labour-intensive and costly, and some places are difficult to access. Consequently, with the advent of techniques including remote sensing (RS), global positioning system (GPS) and geographical information system (GIS), many researches are increasingly relying on the classification of RS images as a means to predict snail distributions. Previous studies have used indices–such as land surface temperature (LST), normalized difference vegetation index (NDVI), and normalized difference water index (NDWI)–extracted directly from RS images as predictors of distributions pattern[5]. These methods can assist in understanding spatial patterns of snail habitats at a broad scale. However, because of poor accuracy and high misclassification error associated with those methods, exploring a variety of new predictive models based on statistical algorithms to predict patterns of snail distribution has been growing rapidly[6,7].

Ecological niche models (ENMs) is a powerful method for producing predictive risk maps of species distribution and has been widely applied to many species in various fields, especially invasive species[8,9]. The concept of “ecological niche” was first proposed by Joseph Grinnell in 1917 to define species distribution patterns (excluding migration)[10]. Based on this concept, ENMs are developed to predict the geographical distribution of species through analysing the statistical relationships between known localities of the species of interest and risk variables. How to compare the relative performance of different models and make the final selection of best modelling strategy remains a challenge[11,12].

**Fig 1:**
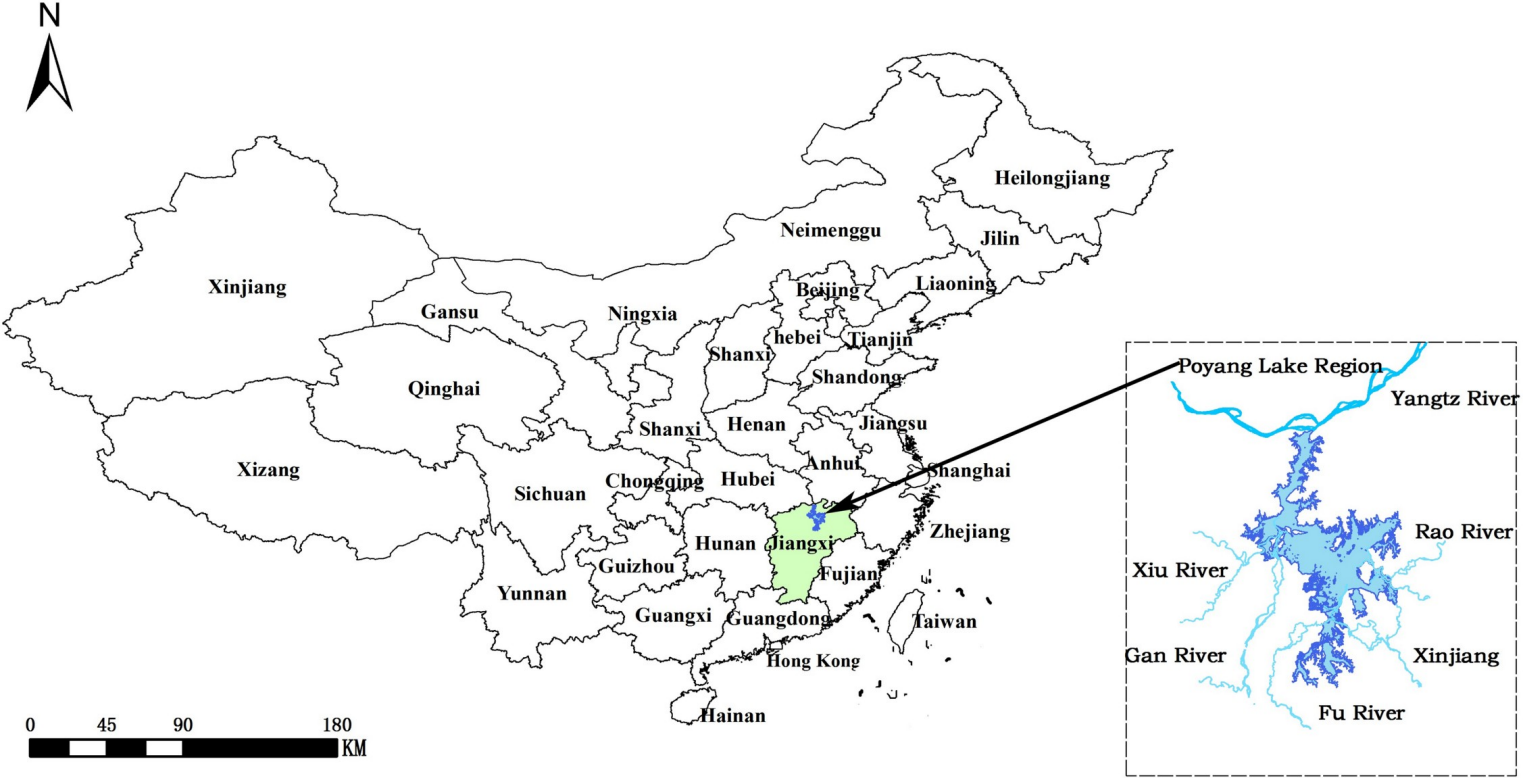
Location of study area to predict high-risk habitats of Oncomelania hupensis, the intermediate host of Schistosoma japonium in the Poyang Lake region of China. Poyang Lake is situated in the northern part of Jiangxi Province and near the southern bank of the middle and lower reaches of Yangtze River. This study covers the core region of the lake with areas of 3,636 km^2^.

Four lake and marshland provinces along the Yangtze River (Hubei, Hunan, Jiangxi and Anhui) are high-prevalence regions for schistosomiasis japonica, accounting for approximately 94% of the infected persons in China[13]. Within this region, Dongting Lake and Poyang Lake regions account for 86% of infections[3]. Therefore, swamp and lake areas are the current major focus for schistosomiasis prevention and control. As the largest freshwater lake in China, Poyang Lake constitutes the largest endemic area for schistosomiasis and undoubtedly is a priority for schistosomiasis control. Poyang Lake is located in northern Jiangxi Province and near the southern bank of the middle and lower reaches of the Yangtze River (Fig 1). Five main tributaries, the rivers Gan, Fu, Xiu, Rao and Xin, flow into Poyang Lake, which empties each year into the Yangtze River via Hukou[14]. During flooding (April to June), water from these five rivers recharge Poyang Lake. The water level peaks from July to September. Thus, flood plains develop during the period April to September. Between November and early March, the water subsides and this area becomes a landscape of marshlands[15]. As a result, Poyang Lake is a typical seasonal water-land transition lake with dramatic fluctuations in water level, historically ranging from 12 m to 17.2 m measured by Xingzi hydrometric station; this corresponds to a change in water surface from about 500 km^2^ in the dry season to up to approximately 3 700 km^2^ (7-fold increase) in the rainy season. Seasonal variations in the water level, vast marshlands as well as an extensive grassland plain, provides an excellent environment for the growth and development of *O*. *hupensis*. To date, of 12 administrative counties (cities or districts) located along Poyang Lake, four (Jinxian, Hukou, Lushan and Gongqing) have reached the national criterion for interruption of Schistosomiasis japonica transmission (schistosoma japonicum infection rate below 1% in humans and bovines, and no acute schistosomiasis in humans found and no schistosome-infected snails detected for two consecutive years), while seven counties (Duchang, Yongxiu, Yugan, Xingzi, Xinjian, Nanchang and Poyang) have reached the national criterion for transmission control (no infected schistosomiasis in humans and bovines, as well as no infected snails found for five consecutive years)[16]. Therefore, Poyang Lake remains a key area of endemic schistosomiasis in China.

In this study, we aimed to identify high-risk snail habitats in the Poyang Lake region using an integrated ecological niche modelling approach, with both presence-absence (PA) and presence-only (PO) models discussed. This study was based on *O*.*hupensis* occurrence data and a range of variables, including remote sensing derived data, biological climate data, soil-related data and economic-related data, to develop a new technical framework for predicting snail habitats. These results can also provide guidance on–and a theoretical basis for–the national surveillance and control of schistosomiasis.

## Methods

### Study area

The extent of potential snail habitats in Poyang Lake depends on the areas with fluctuation of water level. Previous studies have shown that about 95% of snail habitats are distributed in the plains (elevation 14 to 17 m); at elevation ≤13 or >17 m, areas are not suitable as snail habitat[17]. The highest (17.16 m elevation on July 25, 2007) and lowest (12.58 m elevation on March 24, 2003) water levels have been measured at Xingzi hydrometric station in Jiangxi Province, which were the corresponding date for the highest and lowest elevation used in this study. The study area covers about 3,636 km^2^.

### Data sources

#### Species data

The data used to construct the models included 3 000 occurrence records of *O*.*hupensis*. A square frame covering an area of 0.11 m^2^ was used to count snails during 2016. This method has been widely applied in China. In each sampled square frame, *O*.*hupensis* were collected by trained local workers, then transferred to the laboratory, and the number of surviving *O*.*hupensis* were recorded[18]. The latitude and longitude coordinates of each occurrence record were obtained by a handheld GPS instrument (MobileMapper10, Thales Navigation, Paris, France). While for the absence records, 3 000 pseudo-absence records were obtained via random extraction from the study area, excluding occurrence record locations.

All the presence and absence data were randomly separated into model training and testing data sets using a 75–25% split, respectively. For the training sets based on PO models, only 75% of the presence data were included.

#### Risk factors

Variables previously confirmed as important determinants of the distribution of snail habitats were chosen to construct the models. The types of variables used in this study are listed and briefly described in Table 1.

**Table 1:** Descriptions and abbreviations of variables used to develop a range of ecological niche models for predicting high-risk habitats of *Oncomelania hupensis*, the intermediate host of *Schistosoma japonium* in the Poyang Lake region of China.

Descriptor type	Variable description (Abbreviation)	Resolution	Variable type
RS	Normalized difference vegetation index (NDVI)	30 m	Continuous
	Land surface temperature (LST)	30 m	Continuous
	Elevation (DEM)	30 m	Continuous
	Aspect (asp)	30 m	Continuous
	Slope(slope)	30 m	Continuous
	Distance to nearest river (water)	30 m	Continuous
Bio-climatic	WorldClim (bio1-bio19)	1 km	Continuous
Soil	Agrotype (soiltype)	1 km	Categorical
	Soil erosion (qinshi)	1 km	Categorical
	Soil texture (sand, silt, clay)	1km	Continuous
Geomorphic type	Plain, hill, mountain, et al. (mor)	1 km	Categorical
Vegetation type	Coniferous、brush、broad leaved forests, et al. (veg)	1km	Categorical
Land use	Water, flat, grass, sand beach (landuse)	30 m	Categorical
Ecosystem type	Wilderness, settlement, wetland, et al. (eco)	1 km	Categorical
Socio-economic	Distribution of GDP (gdp)	1 km	Continuous

Normalized difference vegetation index (NDVI), land surface temperature (LST), and distance to the nearest water body were extracted from remote sensing images from Landsat 7 ETM+ on March 24, 2003, which were downloaded from Geospatial data cloud (http://www.gscloud.cn/). The layer of DEM was obtained from China resource and environment data cloud platform (http://www.resdc.cn/) and ArcGIS 10.0 software was used to extract the indices of slope and aspect from the DEM layer. All the RS indices have the same spatial resolution of 30m.

Climatic data were obtained from Worldclim version 1.4 (http://www.worldclim.org/), a set of global climate layers including 19 bioclimatic variables with a spatial resolution of 1km^2^ (as summarized in S1 Table) such as average monthly climate data for minimum, mean, and maximum temperature as well as precipitation, representing annual trends, seasonality and extremes of environment factors. To avoid multi-collinearity among variables and reduce the model complexity, an approach to reduce dimensionality was applied. A pairwise correlation matrix was constructed for these 19 variables and a correlation coefficient >0.7 was considered to be high. For each variable, the differences between snail presence and absence datasets were compared by *t*-tests. For highly correlated pairs of variables, the one which had relatively less biological meaning and a smaller difference between presence and absence datasets was removed. Finally, 7 variables (Bio3, Bio4, Bio5, Bio9, Bio10, Bio15, Bio17) were selected to describe temperature and precipitation, rather than the original 19 variables.

Another 6 remote-sensing datasets with spatial resolution of 1km in 2015 describing soil, geomorphology, vegetation, land-use, ecosystem and socio-economics, were provided by Data Center for Resources and Environment Sciences, Chinese Academy of Sciences (RESDC) (http://www.resdc.cn/).

### Data processing

All maps were rescaled to spatial resolution of 30m to conform to the data structure using the resampling technique in ArcGIS 10.0. The nearest neighbor method was used to resample categorical variables and the approach of bilinear interpolation was applied instead for continuous variables.

In addition, the masking technique was also applied in ArcGIS 10.0 to extract exactly the same geographic extent of all maps. Considering suitable elevation for snail habitat (14–17m), we selected the geographic boundary of Poyang Lake on July 25, 2007 with elevation of 17.16m as the mask to obtain the largest and potential snail habitat areas.

### Models development and assessment

The number of ENMs available for predicting species distribution is immense and can be classified into two categories based on the data types used for model construction. Predictive maps produced only based on species’ occurrence data are categorized as presence-only models (hereafter “PO” models) and those requiring both presence and absence data are classified as presence-absence models (hereafter “PA” models). Four PO models (Maximum Entropy Models (MAXENT), Genetic Algorithm for rule-set Production (GARP), Bioclim and Domain) and six PA models (Generalized Linear Models (GLMs), Multivariate Adaptive Regression Splines (MARS), Flexible Discriminant Analysis (FDA), as well as machine algorithmic models Random Forest (RF), Classification Tree Analysis (CTA), Generalized Boosted Model (GBM)) were constructed. See S1 Text for details on using these models.

Predictions from each model were compared to each other based on three indices, receiver-operating characteristic curve (ROC curve), Kappa and calibrating plot. Firstly, ROC graphing was applied to evaluate the discrimination performance of these models. Discrimination performance is the ability of a model to correctly distinguish occupied from unoccupied sites, which can be measured by setting a threshold and predicting the species to be present or absent at a site based on whether the model prediction is above or below the threshold. The most common measure of discrimination is the area under the ROC curve (AUC). ROC curve is a two-dimensional graph, with true positive rate (sensitivity) as Y axis and false positive rate (1–specificity) as X axis; it can be used to illustrate the relationship between sensitivity and specificity. Secondly, Cohen’s kappa, which is also called consistency test, was adopted to visualize a model’s consistency. The kappa statistic defines the accuracy that might have resulted by chance alone. It ranges from –1 to +1, where +1 indicates perfect agreement between prediction and observation, and –1 indicates complete disagreement. In addition, values of 0 or less indicate agreement no better than random classification. To enable a standardized evaluation of all models, threshold values (Maximized value of the kappa; Maxkappa) were applied to transform all predictions to binary (presence-absence) predictive maps. Finally, our calibration plot compares the agreement between predicted probability of occurrence and observed proportions of sites occupied. It shows the ability of the model to make unbiased estimates of the probability of the outcome[19]. The calibration plot can be developed by breaking the predicted probabilities up into several intervals, and plotting the proportion of evaluation sites that are observed to be occupied within each of these intervals against the median predicted value of each interval. When the points lie closely along a 45° line then the model is well calibrated and has good agreement[20].

Based on the comprehensive assessment of results, the model with an ROC curve value >0.8, kappa value >0.5 and relative good calibration was defined as superior models. Superior models were used to build the following ensemble model and the variable importance for detecting snail habitat were further evaluated. The importance of each variable was estimated using a permutation procedure.

### Ensemble model

Models with superior predictive accuracy were combined together to construct an ensemble model to generate the final predicted risk map of snail habitats through the method of AUC-based weighted average[21,22].

1. Unify every predictive map by normalization, so that the value of each grid cell ranged from 0 to 1.
2. All the grid layers after normalization were weighted respectively on the basis of its weighted AUC score. The equation used was as follows,
  $$y_{j}=\sum_{i=1}^{n}w_{i}x_{ij}$$ (1)
  $$w_{i}=\frac{r_{i}}{\sum_{m=1}^{n}r_{m}}$$ (2)
  *x*_*ij*_ is the value of grid *j* in the predictive map generated by model *i*; *y*_*j*_ is the snail habitat probability of grid *j* in the final map generated by the ensemble model; *y*_*j*_ ranges from 0 to 1, and the value of 1 indicates the highest possibility of snail habitat occurrence; n is the count of the individual models used to construct the ensemble model; *w*_*i*_ is model’s weighted score; *r*_*i*_ is model’s AUC value.
3. All the grid layers after these two steps above were imported to ArcGIS10.0 to generate the final ensemble model, which was evaluated independently through field investigations.

## Results

### Overall accuracies of models

**Fig 2:**
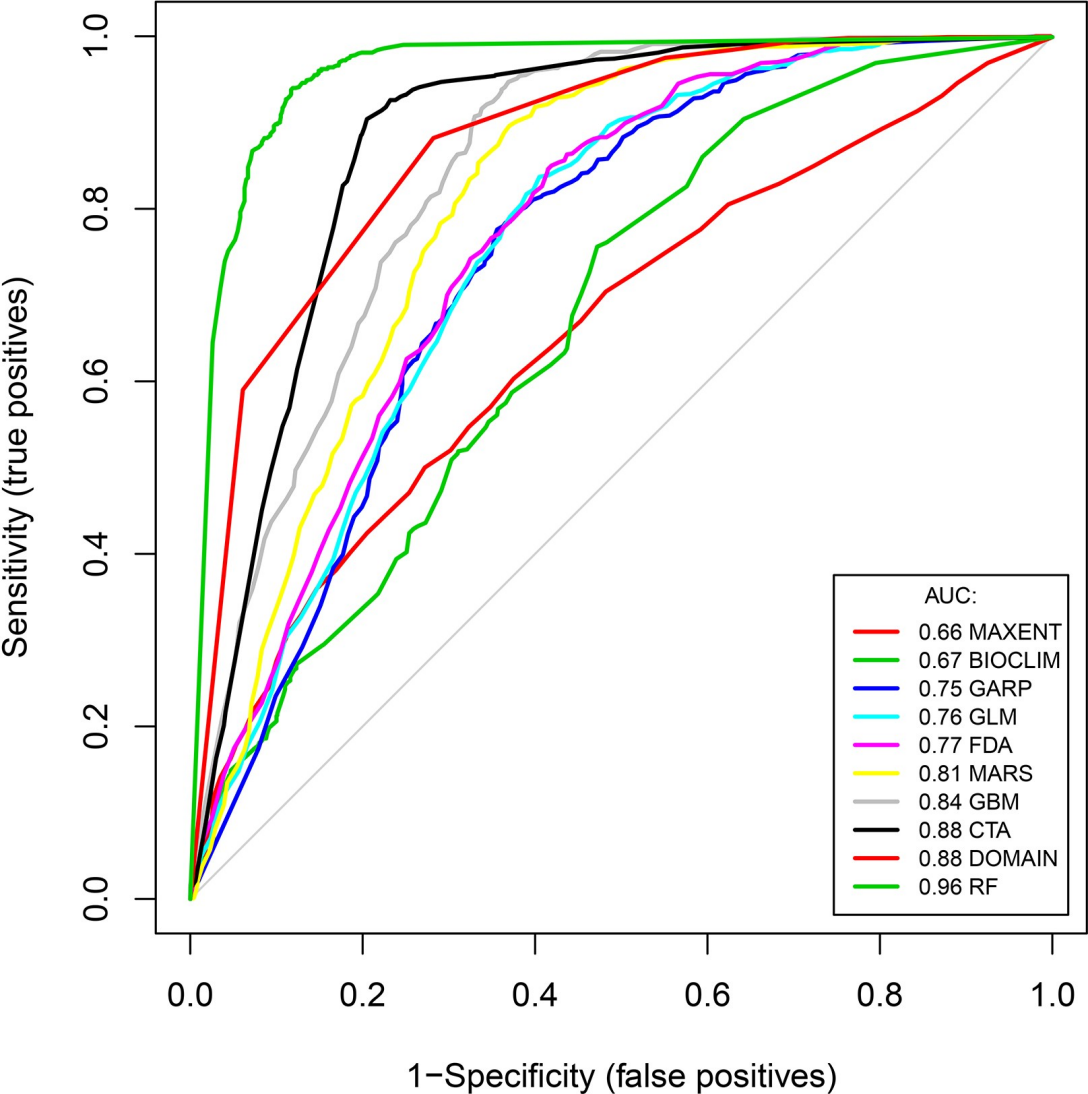
The average area under the receiver-operator characteristic (ROC) curve for all ecological niche models for predicting high-risk habitats of Oncomelania hupensis, the intermediate host of Schistosoma japonium in the Poyang Lake region of China. AUC value for RF (AUC = 0.96) was highest among current models. Discriminative ability was relative better for MARS, GBM, CTA and Domain (AUC = 0.81, 0.85, 0.88 and 0.88, respectively). However, MAXENT and Bioclim models showed poorest discrimination with AUC<0.7. The average AUC for the six PA models was 0.84, outperforming the four PO models (AUCmean = 0.74).

ROC curves indicated that the ten model’s performance varied markedly from each other in their predictive ability (Table 2 and Fig 2). Discriminative ability of RF (AUC = 0.96) was best among current models and its variance was lowest (AUC.sd = 0.004). Discriminative ability was relative better for MARS, GBM, CTA and Domain (AUC = 0.81, 0.85, 0.88 and 0.88, respectively). However, MAXENT and Bioclim models showed poorest discrimination with AUC<0.7. The average AUC for the six PA models was 0.84, outperforming the four PO models (AUC_mean_ = 0.74).

**Table 2:** Estimated accuracies of ecological niche models for predicting high-risk habitats of *Oncomelania hupensis*, the intermediate host of *Schistosoma japonium* in the Poyang Lake region of China. AUC–area under the curve; AUC.sd–standard deviation of AUC; Maxkappa–maximum value of the kappa statistic for agreement; kappa.sd–standard deviation of the kappa statistic.

Model	AUC	AUC.sd	Maxkappa	kappa.sd
MAXENT	0.655	0.012	0.224	0.022
BIOCLIM	0.674	0.012	0.284	0.021
GARP	0.748	0.011	0.420	0.020
GLM	0.757	0.011	0.432	0.020
FDA	0.767	0.011	0.431	0.020
MARS	0.807	0.010	0.526	0.018
GBM	0.845	0.009	0.575	0.018
DOMAIN	0.877	0.007	0.600	0.018
CTA	0.879	0.008	0.699	0.016
RF	0.962	0.004	0.821	0.013

The trends observed in kappa were generally similar to those assessed with AUC (Table 2). RF–which had the highest discriminative ability as assessed by AUC–also had the highest kappa scores (0.82). Consistency as assessed by Kappa was generally higher for CTA and Domain than GBM, MARS, FDA, GLM as well as GARP, and poorest for MAXENT and Bioclim among current models. The mean kappa value for PA models (0.58) was also higher than that for PO models (0.38).

**Fig 3:**
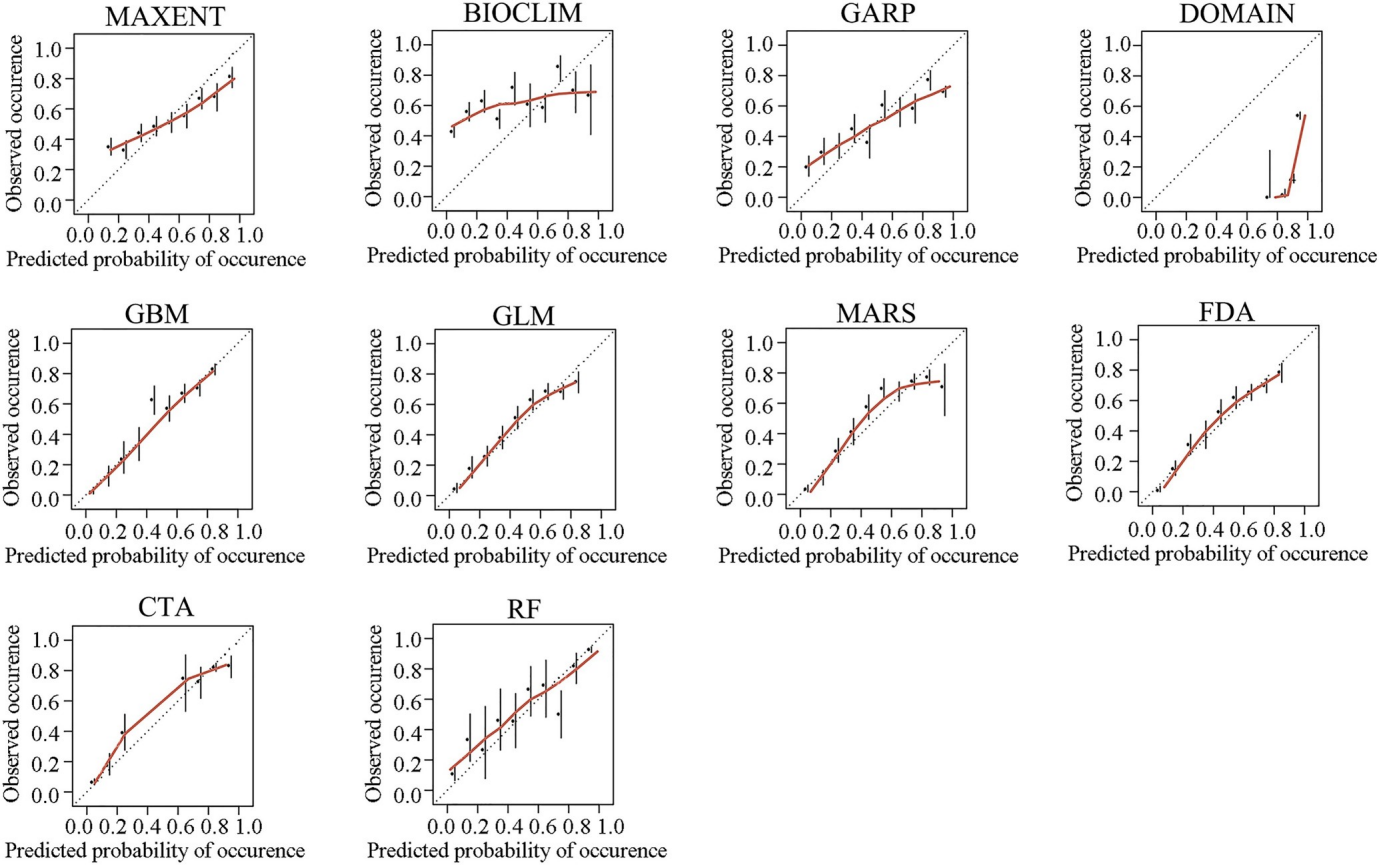
Calibration plot from all ecological niche models for predicting high-risk habitats of Oncomelania hupensis, the intermediate host of Schistosoma japonium in the Poyang Lake region of China. Models including GLM, FDA, CTA and GBM were relatively better calibrated–calibration plots nearing the 45° diagonal. In addition, models of MAXENT, GARP, Bioclim and Domain were poorly calibrated.

For the results of calibration plots and goodness-of-fit tests (Fig 3), models including GLM, FDA, CTA and GBM were relatively better calibrated–calibration plots nearing the 45° line. In addition, MAXENT, GARP, Bioclim and Domain models were poorly calibrated.

Taken together, RF, CTA, GBM and MARS showed superior accuracy among these ten Ecological Niche Models constructed in this study.

### Variables importance

Four models (RF, CTA, MARS and GBM) with superior accuracy were used to construct the final ensemble model and assess the importance of variables.

To evaluate the final ensemble model, another small dataset, including 822 absence records and 178 presence records, was manually collected in the field during 2016 for external validation. Its AUC was 0.89, sensitivity was 0.79 and specificity was 0.82.

**Fig 4:**
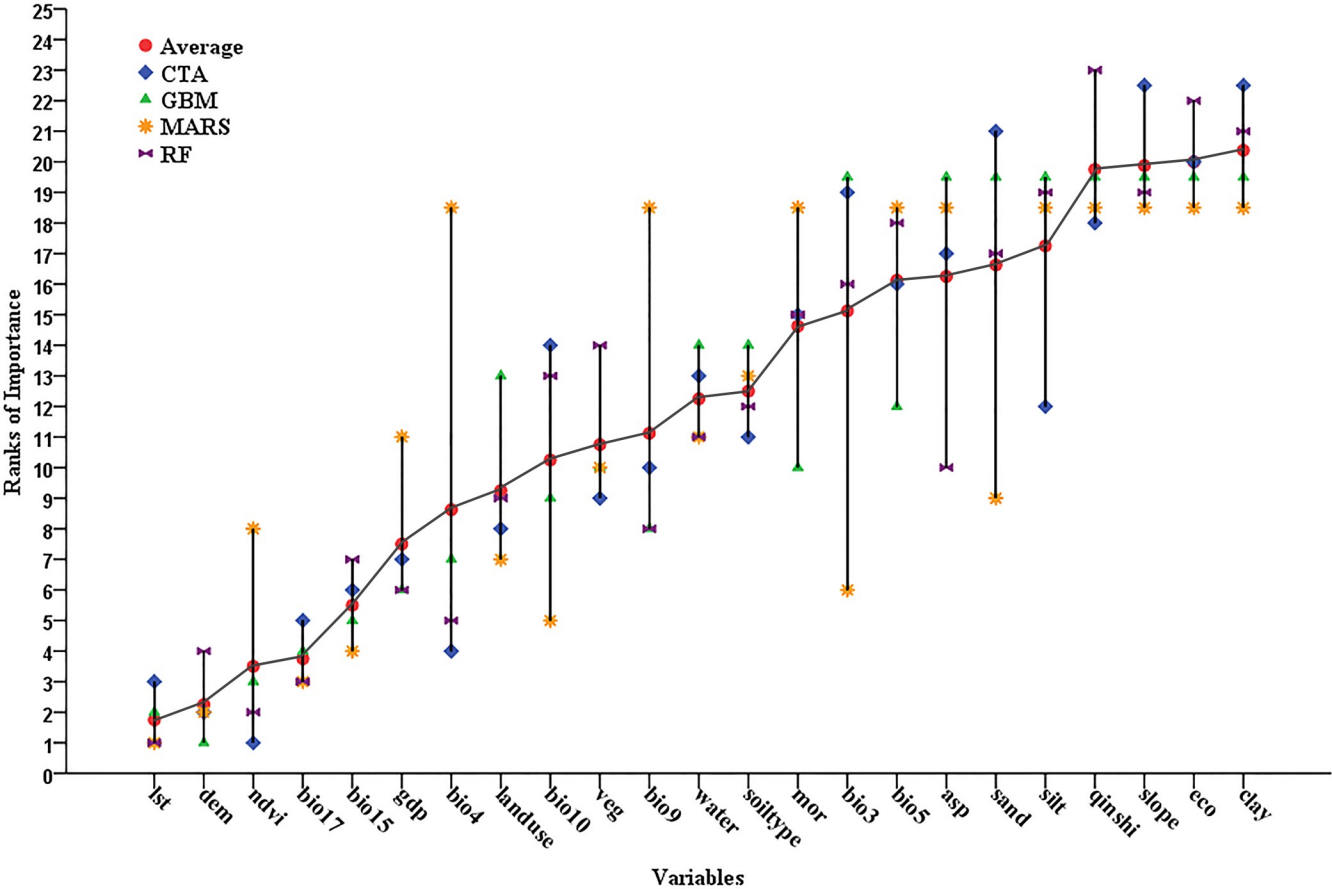
Ranks of importance of variables affecting predictive performance of four ecological niche models (CTA, GBM, MARS and RF) for predicting high-risk habitats of Oncomelania hupensis, the intermediate host of Schistosoma japonium in the Poyang Lake region of China. The vertical bar indicates the importance rank of each variable contributing to four models (CTA, GBM, MARS, and RF, respectively), and the horizontal bar shows the average importance rank of each variable. The smaller the latter value, the more important is the variable. LST, DEM and NDVI were found to be the most important factors influencing these four models.

As shown in Fig 4, the vertical bar indicates the importance rank of each variable contributing to these four models, and the horizontal bar shows the average importance rank of each variable. The smaller the latter value, the more important is the variable. LST, DEM and NDVI were found to be the most important factors influencing these four models, making the highest contribution. Other environmental factors that significantly influenced the model were Bio17, Bio15, Bio4 and landuse, which were amongst the top ten important determinants of the geographical distribution of snail habitats. It was noted that the level of GDP also showed a relatively high contribution to these predictive models.

### Predictive map of high-risk snail habitat

**Fig 5:**
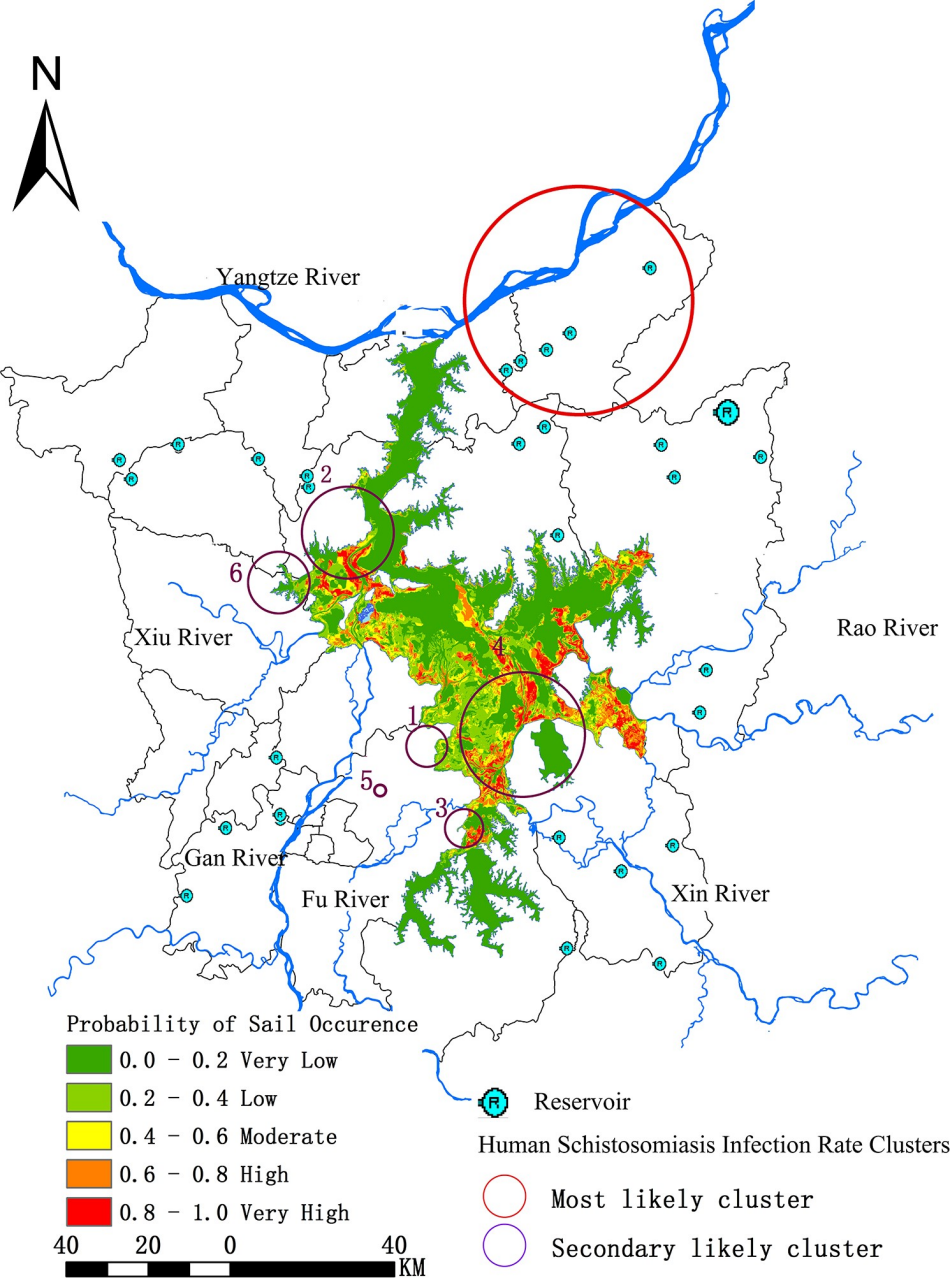
Predictive risk map of habitats of Oncomelania hupensis, intermediate host of Schistosoma japonium in the Poyang Lake region, and high-risk schistosomiasis clusters. **Risk is expressed as probability of snail occurrence.** The high-risk snail habitat areas were classified into four grades in terms of urgency for prevention and control actions. Of which, the highest risk level and most significant areas were very high risk snail habitat within schistosomiasis clusters, mainly distributed in the watershed of the River Fu in Yugan County as well as the watershed of the River Gan and the River Xiu in Xingzi County, covering an area of 52.3 km^2^.

The predictive risk map for snail habitats in Poyang Lake region generated by the above ensemble model was expressed as a probability (Fig 5). Probability of the presence of snail habitats ranged from 0 to 1, with a color closer to red indicating climatic, environment and economic factors that are more suitable for the growth or reproduction of snails. The distribution risk of potential snail habitats in Poyang Lake region were classified into five grades (very low, low, moderate, high and very high) with areas of 2 096.1 km^2^, 640.1 km^2^, 371.2 km^2^, 322.5 km^2^ and 206.9 km^2^, respectively, where population at risk were 136 300, 53 900, 30 600, 11 700 and 3 800, respectively. The area most suitable for the presence of snail habitats are located in: (1) middle part of Xingzi County through which the River Xiu and the River Gan flow; Yugan County (the River Fu and the River Xin); Poyang County (the River Rao) and the north-east part of Poyang County; (2) intersection of Xinjian, Yongxiu and Duchang counties; (3) other high-risk areas scattered along the peripheral regions of Poyang Lake.

Overlaid with our previous results on identified high-risk clusters of schistosomiasis [14], the final high-risk snail habitat map was generated and the area was classified into four grades in terms of urgency for prevention and control actions (Fig 5 and Table 3). The highest risk level and most significant areas were very high risk snail habitat within schistosomiasis clusters, which are mainly located in Cluster 4 and Cluster 2, as well as partly in Cluster 3, covering an area of 52.3 km^2^. Areas belonging to the first grade were mainly distributed in the watershed of the River Fu in Yugan County as well as the watershed of the River Gan and the River Xiu in Xingzi County. The second grade was high and moderate-high risk snail habitat within schistosomiasis clusters, which were located partly in Xingzi County and in most regions in the middle part of Yugan County, covering areas of 72.8 km^2^ and 79.7 km^2^, respectively. The third grade was very high risk snail habitat outside schistosomiasis clusters, covering an area of 154.6 km^2^. The fourth grade was high and moderate risk snail habitat outside schistosomiasis clusters, covering areas of 249.7 km^2^ and 291.5 km^2^, respectively.

**Table 3:** Classification of areas ranked by priority for prevention and control of high-risk habitats of *Oncomelania hupensis*, the intermediate host of *Schistosoma japonium* in the Poyang Lake region of China.

Grades	Description	Coverage Areas
First	very high risk snail habitats within schistosomiasis clusters	52.3km^2^
Second	high and moderate high-risk snail habitats within schistosomiasis clusters	152.5km^2^
Third	very high risk snail habitats outside schistosomiasis clusters	154.6km^2^
Fourth	high and moderate risk snail habitats outside schistosomiasis clusters	541.2km^2^

## Discussion

As the largest freshwater lake and typical marshland areas, Poyang Lake region is widely known as a suitable site for endemic schistosomiasis and is a major concern for schistosomiasis control in China. This study has attempted to predict high-risk snail habitats in the Poyang Lake region by ecological niche modelling to provide assistance for future targeted monitoring and control. This study presents predictive maps of the spatial distribution of *Oncomelania hupensis*, the key intermediate host snail of schistosomiasis, which is vital for long-term sustainable effective schistosomiasis control in the Poyang Lake region.

The MAXENT model showed the worst predictive performance in our study, which is different from previous reports by Scholte et al[6], who predicted the geographical distribution of *Schistosomiasis mansoni*’s intermediate host and claimed that MAXENT has high accuracy. The MAXENT model was sensitive to sampling errors, which might easily introduce a bias to modeling. The GARP model can be greatly influenced by selection of the training data set and has large uncertainties because of different results for each computation. Predictive results of the GARP model are always a discrete value of 0 and 1, hence showed a weak predictive accuracy. The Bioclim model could be considered as the simplest model in our study since it just assumes a rectilinear environment envelope; it does not include correlations or interactions between factors[12]. This study showed that the Bioclim model showed relatively poor performance. Similar results have been reported in many studies predicting the geographical distribution of multi-species using Bioclim and those studies have reported poor results[12]. Domain model was found to have excellent discrimination ability but weak calibration, which agrees with previous studies[23]. Similar to Bioclim, Domain is also based on an environment envelope, but it has the ability to include discontinuity of the species’ records. Therefore, excellent discrimination ability of Domain model might be due to intense spatial auto-correlation in snails’ presence records. However, Domain model also has a major limitation: for each potential site, only the nearest neighbor point within the envelope is used to determine its suitability for species, which might result in weak calibration. Most parameters for the four PO models were maintained at their default settings because of a lack of reasonable prior information, which might have introduced some subjectivity and thus bias.

Of the six presence-absence models used in this study, GBM, CTA and RF were based on machine learning algorithms of classification trees, desirable to handle unordered, nonlinear and multidimensional data. They produced relatively higher accuracy compared to other models used in our study. RF, especially, showed the best predictive performance and could predict the geographical distribution of snail habitats accurately. We also found that these six PA models differed from each other in their complexity. RF can be considered the most complex: it fits many classification trees to a data set, and then combines the predictions from all the trees. In our study, it showed the highest predictive accuracy. CTA and GBM are also relatively less complex than the other models. Hence, it seems that increased model complexity may contribute to better predictive performance. For MARS, it showed relatively good accuracy, indicating that there might be non-linear correlation between snail habitats and risk variables, and the variables might have complicated associations among each other, which explains why classical statistical models such as GLMs only have normal predictive performance.

With regard to the prediction of snail habitats, we found that the overall predictive performances of PA models–especially machine learning algorithm based approaches–outperformed PO models. This is consistent with the results obtained by Brotons[24] et al., who proposed that absence data could help to identify low suitability areas that might have otherwise been classified as good habitats if only presence data were used.

This study also confirmed that snail habitats are related to multiple factors including several ecological environment variables and social-economic variables. LST, DEM and NDVI were the top three variables affecting snails’ geographical distribution. Similar results have also been reported in many previous studies[25,26]. Meanwhile, the indexes of precipitation–including Bio17 and Bio15 –also showed relatively higher importance. These important environment factors ultimately affect soil moisture content and vegetation density to influence the growth and reproduction of snails. In addition, social-economic index of GDP was also shown to have high contribution to the predictive ability of the model. The economic development of Poyang Lake region has been quite unbalanced; undeveloped counties-including Jiujiang, Hukou, Pengze, Xingzi, Poyang, Duchang and Yugan-belong to traditional agricultural regions with quite a low level of mechanization. Most local residents in these regions live by farming, fishing or grazing livestock. Considering that livestock from free-range farming is an important infection source for schistosomiasis, areas with a traditional pattern of farming and grazing–especially in economically undeveloped regions–should be a focus for schistosomiasis control.

The high-risk areas of both snail habitats and schistosomiasis were found to be mainly concentrated in the confluence of Poyang Lake and five main tributaries, which have a high risk of schistosomiasis epidemics and require significant attention. One potential explanation is that these areas appear frequently to alternate between land and water in different seasons, providing an ideal environment for snail growth and reproduction. Furthermore, most of the local residents in these areas live by farming or fishing, which increases their contact with contaminated water. Xingzi, in particular, is an undeveloped county, and the majority of infected individuals in this county are farmers or fishermen. They become infected mostly due to the behaviors of grazing or fishing[14]. The classification of prevention and control priority levels for high-risk snail habitats, as shown in Fig 5, could provide precise guidance for local authorities to formulate an effective targeted control strategy. The primary and most urgent for prevention and control of both schistosomiasis and snails is the first grade, which is mainly distributed in the watershed of the River Fu in Yugan County as well as the watershed of the River Gan and the River Xiu in Xingzi County. This region has a very high risk of both schistosomiasis and snails; hence it has a high prevalence of infected snails. The epidemic conditions for infection and transmission in this region might be very easily to meet, therefore it has a high spread risk of schistosomiasis, even outbreaks. Therefore, areas of first grade should be prioritized for schistosomiasis prevention and control. Meanwhile, other grades also should be paid attention based on the available health resources. However, it should be noted that so-called “high-risk sites” in our study represent “suitable locations for the snail habitats”; that does not mean the snails will always be present at the predicted sites.

One issue deserves a discussion. Data resolutions were different in the original variables used, which might affect predictive accuracy somewhat, especially variable importance due to the scale transformation. It would be informative to explore whether and in what degree different scale and scale changes impact the predictive accuracy. In addition, variables used to construct the models in our study came from different years, which might be a limitation for this kind of study of habitat identification. However, the spatial distribution of variables e.g. land use and vegetation type did not show large changes during shorter time periods in Poyang Lake area, and therefore was unlikely to have a great influence on the study results. Furthermore, all the absence records for the process of modelling building were generated from background areas of the study extent without field validation although we checked them via the techniques of visual interpretation of remote sensing images, which might introduce some uncertainties. But we did validate the final model’s performance with field investigated data, including both the presence and absence records of snail habitats.

In summary, this study depicted the spatial distribution of snail habitats in the Poyang Lake region through diverse ecological niche models and compared their performance in terms of predictive accuracies. The results showed that the overall predictive performance of presence-absence models outperformed presence-only models in prediction of snail habitats. Furthermore, increasing model complexity might contribute to predictive accuracy and models based on machine learning algorithms of classification trees present higher accuracy than others. Prediction distribution of snail habitats can provide precise guidance and a theoretical basis for the targeted surveillance and control of schistosomiasis in the Poyang Lake area. The high-risk areas of both snail habitats and schistosomiasis are mainly concentrated in the confluence of Poyang Lake and five main tributaries, which have a high risk of schistosomiasis epidemics and require significant attention. Among these, the most serious and significant areas were distributed in the watershed of River Fu in Yugan County as well as the watershed of River Gan and River Xiu in Xingzi County.

## Supporting information

S1 Table Description of bio-climatic variables (Worldclim:bio1-bio19) used in the process of modelling. (DOCX)

S1 Text Detailed explanations of the PO and PA models used in this study. (DOCX)
