# Supplementary material for: Identification of high-risk habitats of Oncomelania hupensis, the intermediate host of schistosoma japonium in the Poyang Lake region, China: A spatial and ecological analysis
Source: PLoS Negl Trop Dis. 2019 Jun 17;13(6):e0007386. doi: 10.1371/journal.pntd.0007386 (PMC6597197; doi:10.1371/journal.pntd.0007386)
Supplement: S1 Table — (DOCX) [file pntd.0007386.s001.docx]

**S1 Table. Description of bio-climatic variables (Worldclim:bio1-bio19) used in the process of modelling.**

| Bio-climatic variables | Variable description |
| --- | --- |
| Bio1 | Annual mean temperature |
| Bio2 | Mean diurnal range (Mean of monthly (max temp - min temp)) |
| Bio3 | Isothermality (BIO2/BIO7) (*100) |
| Bio4 | Temperature seasonality (standard deviation *100) |
| Bio5 | Max temperature of warmest month |
| Bio6 | Min temperature of coldest month |
| Bio7 | Temperature annual range (BIO5-BIO6) |
| Bio8 | Mean temperature of wettest quarter |
| Bio9 | Mean temperature of driest quarter |
| Bio10 | Mean temperature of warmest quarter |
| Bio11 | Mean temperature of coldest quarter |
| Bio12 | Annual precipitation |
| Bio13 | Precipitation of wettest month |
| Bio14 | Precipitation of driest month |
| Bio15 | Precipitation seasonality (Coefficient of Variation) |
| Bio16 | Precipitation of wettest quarter |
| Bio17 | Precipitation of driest quarter |
| Bio18 | Precipitation of warmest quarter |
| Bio19 | Precipitation of coldest quarter |
