# Supplementary material for: Identification of high-risk habitats of Oncomelania hupensis, the intermediate host of schistosoma japonium in the Poyang Lake region, China: A spatial and ecological analysis
Source: PLoS Negl Trop Dis. 2019 Jun 17;13(6):e0007386. doi: 10.1371/journal.pntd.0007386 (PMC6597197; doi:10.1371/journal.pntd.0007386)
Supplement: S1 Text — (DOCX) [file pntd.0007386.s002.docx]

**S1 Text. Detailed explanations of the PO and PA models used in this study.**

***PO models***

Two PO models (Bioclim and Domain) were constructed by using the software DIVA-GIS version 7.5 (University of California, USA, http://www.diva-gis.org/download).

For the MAXENT model, MAXENT version 3.3.3k (Princeton University, USA, <http://biodiversityinformatics.amnh.org/open_source/maxent/>) was used. This program only needs presence data as input, together with coordinate data for each record and environmental layers. The output format of “logistic” was selected and Jackknife test was chosen to estimate the importance of the environmental variables. The other parameters used the default values of the program.

GARP is non-deterministic and every run using the same data will produce slightly different results. GARP version 1.1.6 (University of Kansas Center for Research, USA, <http://www.nhm.ku.edu/desktopgarp/index.html>) was used to construct the model. We set GARP to perform 100 runs with a convergence limit of 0.01 and 1 000 maximum iterations. All four rule types (atomic, range, negated range and logistic regression) were employed as well as the best subset feature of GARP. We then used the summation feature in the ArcGIS Raster Calculator to make a final, cumulative predictive map.

***PA models***

The Biomod 2 (Biodiversity modelling) package of R software version 3.3.3 (Bell laboratories, USA, <https://www.r-project.org/>) were used to perform the PA models, including RF, CTA, GBM, FDA, GLMs and MARS.

Visualization and geostatistical display of the results were carried out in ArcGIS version 10.0 (ESRI; Redlands, USA). The final models’ results were plotted with a 30m spatial resolution.
